# Supplementary material for: Differential effects of 40S ribosome recycling factors on reinitiation at regulatory uORFs in GCN4 mRNA are not dictated by their roles in bulk 40S recycling
Source: Commun Biol. 2024 Sep 4;7:1083. doi: 10.1038/s42003-024-06761-x (PMC11375166; doi:10.1038/s42003-024-06761-x)
Supplement: Supplementary file 1 — Supplementary Information [file 42003_2024_6761_MOESM1_ESM.pdf]

SUPPLEMENTARY INFORMATION

Differential effects of 40S ribosome recycling factors on reinitiation at regulatory uORFs in *GCN4* mRNA are not dictated by their roles in bulk 40S recycling

Kristína Jendruchová[1,2#], Swati Gaikwad[3#], Kristýna Poncová[1], Stanislava Gunišová[1], Leoš Shivaya Valášek[1*], and Alan G. Hinnebusch[3*]

[1]Laboratory of Regulation of Gene Expression, Institute of Microbiology of the Czech Academy of Sciences, Videnska 1083, 142 20, Prague, Czech Republic

[2]Faculty of Science, Charles University, Albertov 6, 128 00 Prague, Czech Republic

[3]Division of Molecular and Cellular Biology, Eunice Kennedy Shriver National Institute of Child Health and Human Development, National Institutes of Health, Bethesda, MD 20892, USA

*Corresponding authors: valasekl@biomed.cas.cz, alanh@mail.nih.gov
[#]These authors contributed equally to this work.

Running title: Tma proteins in reinitiation and ribosome recycling

Keywords: Tma, MCTS1, DENR, eIF2D, reinitiation, ribosome recycling

SUPPLEMENTARY TABLES

Supplementary Table 1. Yeast strains used in this study.

This table lists all yeast strains employed in this study along with their respective parental strains used to generate them. The details of strain constructions are described in MATERIALS AND METHODS.

**Name:**	**Genotype:**	**Parental strain:**	**Source:**
YSG142 (WT BY4741)	*MATa his3Δ1 leu2Δ0 met15Δ0 ura3Δ0*	-	Euroscarf
YSG178 (*Δtma64*)	*MATa his3Δ1 leu2Δ0 met15Δ0 ura3Δ0 ydr117CΔ:: kanMX4*	YSG142	This study
YSG181 (*Δtma20*)	*MATa his3Δ1 leu2Δ0 met15Δ0 ura3Δ0 yer007C-AΔ::kanMX4*	YSG142	Euroscarf
YSG184 (*Δtma22*)	*MATa his3Δ1 leu2Δ0 met15Δ0 ura3Δ0 yjr014WΔ::kanMX4*	YSG142	Euroscarf
YSG196 (*Δtma20 Δtma64*)	*MATa his3Δ1 leu2Δ0 met15Δ0 ura3Δ0 yer007C-AΔ::kanMX4 ydr117CΔ::natNT2*	YSG181	This study
YKJ3 (*Δtma20 Δtma22 Δtma64*)	*MATa his3Δ1 leu2Δ0 met15Δ0 ura3Δ0 yer007C-AΔ::kanMX4 ydr117CΔ::natNT2 yjr014WΔ::hphNT1*	YSG196	This study
YKJ6 (*Δtma20 Δtma22*)	*MATa his3Δ1 leu2Δ0 met15Δ0 ura3Δ0 yer007C-AΔ::kanMX4 yjr014WΔ::natNT2*	YSG181	This study
BY4741	*MATa his3Δ1 leu2Δ0 met15Δ0 ura3Δ0*	-	
H4520	*MATa his3Δ1 leu2Δ0 met15Δ0 ura3Δ0 ydr117CΔ::hygMX4*; *yer007C-AΔ::kanMX4*	BY4741	[1]

Supplementary Table 2. Primers used in this study.
This table lists all primers used in this study.

Primer:	Sequence:
SG295	GAGAGTTGACCAATTACCTGACAGT
SG294	CTTGTAGCAAAGATTGGAAAAAGAG
SG296	ACTTGCACCATGTACATCAATTCTA
SG325	CTACTGAATAACCGACTCAATAGATTAGTGTAGCGCAGGATTAGTA CAGCTCTATAGAACGCGGCCGCCAG
SG326	GCTTTGATGTCGGGCATTTTTACGCATTTAAACATTTATATGATATAA ATCACTATAGGGAGACCGGCAG
KJ1	CCCAAGGAAACAGTTCAAGAGCTAAACTAAAGAAAAGCATATTGCA TAAACTATAGAACGCGGCCGCCAG
KJ2	GTAAAAAGTCCTTTTCTCCCAGAACGGTGCTATTACATATTTATGGA TTGCCACTATAGGGAGACCGGCAG
PB238	GCA GCG AGG AGC CGT AAT
KJ27	AGGGCATCGGTCGACGGGGAATAAAG
KJ24 TGG	TTGACAGAAAGGTAACCGTTACCAAAACATC
KJ25 TTG	ATTTGACAGAAAGGTAACCGTTACAAAAACATC
KJ26 ATT	TTTGACAGAAAGGTAACCGTTAAATAAACATCTTG
KJ73 AAA	AGAAAGGTAACCGTTATTTAAACATC
KJ74 AAT	AGAAAGGTAACCGTTAATTAAAC
KJ75 AAG	CAGAAAGGTAACCGTTACTTAAA
KJ76 TAT	ACAGAAAGGTAACCGTTAATAAAACATC
KJ77 CCA	AGAAAGGTAACCGTTATGGAAACA
KJ78 CAA	CAGAAAGGTAACCGTTATTGAAAC A
KJ79 GAC	AGAAAGGTAACCGTTAGTCAAACAT
KJ80 GCT	AGAAAGGTAACCGTTAAGCAAACAT

SUPPLEMENTARY REFERENCES

1. Young DJ*, et al.* Tma64/eIF2D, Tma20/MCT-1, and Tma22/DENR Recycle Post-termination 40S Subunits In Vivo. *Mol Cell* **71**, 761-774 e765 (2018).
