# Supplementary material for: Differential effects of 40S ribosome recycling factors on reinitiation at regulatory uORFs in GCN4 mRNA are not dictated by their roles in bulk 40S recycling
Source: Commun Biol. 2024 Sep 4;7:1083. doi: 10.1038/s42003-024-06761-x (PMC11375166; doi:10.1038/s42003-024-06761-x)
Supplement: Supplementary file 2 — Description of Additional Supplementary Materials [file 42003_2024_6761_MOESM2_ESM.pdf]

Description of Additional Supplementary Files

File name: Supplementary Data 1

Description: Source data behind Figures 3-7

File name: Supplementary Data 2

Description: Plasmids used in this study

File name: Supplementary Data 3

Description: Synthetized DNA inserts used in this study.
