# Supplementary material for: Differential effects of 40S ribosome recycling factors on reinitiation at regulatory uORFs in GCN4 mRNA are not dictated by their roles in bulk 40S recycling
Source: Commun Biol. 2024 Sep 4;7:1083. doi: 10.1038/s42003-024-06761-x (PMC11375166; doi:10.1038/s42003-024-06761-x)
Supplement: Supplementary file 4 — Supplementary Data 2 [file 42003_2024_6761_MOESM4_ESM.docx]

**Supplementary Data 2. Plasmids used in this study.**

This table lists all plasmids used in this study, with construction details described in MATERIALS AND METHODS.

| **Plasmid:** | **Description:** | **Tma-dependence** |
| --- | --- | --- |
| p227 (uORF-less) | low copy *URA3* *GCN4-lacZ* plasmid containing the uORF-less *GCN4* leader created by point mutations in uORF1 (*Hind*III), uORF2 (*Eco*RI), uORF3 (*Kpn*I) and uORF4 (*Bgl*II) ^1^ | N/A |
| pFA6a-KanMX4 | pFA6 backbone containing *KanMX4* resistance marker-based deletion cassette^2^ | N/A |
| pZC3 | pFA6 backbone containing *hphNT1* resistance marker-based deletion cassette^3^ | N/A |
| pZC4 | pFA6 backbone containing *natNT2* resistance marker-based deletion cassette^3^ | N/A |
| **uORF4 constructs** | | |
| pKJ59 | p226 with uORF4 penultimate codon mutated to TAT | Hyperdependent |
| pKJ34 | p226 with uORF4 penultimate codon mutated to TTG | Hyperdependent |
| pKJ35 | p226 with uORF4 penultimate codon mutated to ATT | Hyperdependent |
| pKJ61 | p226 with uORF4 penultimate codon mutated to AAT | Hyperdependent |
| pKJ57 | p226 with uORF4 penultimate codon mutated to CAA | Hypodependent |
| pKJ58 | p226 with uORF4 penultimate codon mutated to GCT | Hypodependent |
| pKJ36 | p226 with uORF4 penultimate codon mutated to TGG | Hypodependent |
| pKJ20 | p226 with uORF4 penultimate codon mutated to TAC | Hypodependent |
| pKJ62 | p226 with uORF4 penultimate codon mutated to CCA | Average |
| pKJ23 | p226 with uORF4 penultimate codon mutated to ATG | Average |
| p226 (uORF4-only) | low copy *URA3* *GCN4-lacZ* plasmid containing *GCN4* leader with uORF4 only at its original position^1^ | Average |
| pKJ21 | p226 with uORF4 penultimate codon mutated to GCG | Average |
| pKJ17 | p226 with uORF4 penultimate codon mutated to CTG | Average |
| **Start-stop constructs** | | |
| pKP76 | low copy *URA3* *GCN4-lacZ* plasmid containing uORF3 only at its original position mutated into a start-stop element | N/A |
| pKP77 | low copy *URA3* *GCN4-lacZ* plasmid containing uORF4 only at its original position mutated into a start-stop element | N/A |
| pKP78 | low copy *URA3* *GCN4-lacZ* plasmid containing uORF1 only at its original position mutated into a start-stop element | N/A |
| pSG194 | low copy *URA3* *GCN4-lacZ* plasmid containing an an uORF1 variant only with coding sequence replaced by the corresponding sequence of uORF2^4^ | N/A |
| **uORF1 constructs** | | |
|  |  |  |
| pSG62 | pSG61 with uORF1 penultimate codon mutated to ATT | Hyperdependent |
| pSG63 | pSG61 with uORF1 penultimate codon mutated to TTG | Hyperdependent |
| pSG64 | pSG61 with uORF1 penultimate codon mutated to AAA | Hyperdependent |
| pSG65 | pSG61 with uORF1 penultimate codon mutated to AAT | Hyperdependent |
| pSG66 | pSG61 with uORF1 penultimate codon mutated to AAG | Hyperdependent |
| pSG67 | pSG61 with uORF1 penultimate codon mutated to TAT | Hyperdependent |
| pSG77 | pSG61 with uORF1 penultimate codon mutated to GCT | Hypodependent |
| pSG78 | pSG61 with uORF1 penultimate codon mutated to CAC | Hypodependent |
| pSG79 | pSG61 with uORF1 penultimate codon mutated to TTT | Hypodependent |
| pSG71 | pSG61 with uORF1 penultimate codon mutated to TGG | Hypodependent |
| pSG72 | pSG61 with uORF1 penultimate codon mutated to TAC | Hypodependent |
| pSG73 | pSG61 with uORF1 penultimate codon mutated to CAA | Hypodependent |
| pSG74 | pSG61 with uORF1 penultimate codon mutated to GAC | Hypodependent |
| pSG75 | pSG61 with uORF1 penultimate codon mutated to GAG | Hypodependent |
| pSG76 | pSG61 with uORF1 penultimate codon mutated to TGT | Hypodependent |
| pSG68 | pSG61 with uORF1 penultimate codon mutated to ATG | Average |
| pSG69 | pSG61 with uORF1 penultimate codon mutated to GCG | Average |
| pSG70 | pSG61 with uORF1 penultimate codon mutated to CTG | Average |
| pSG80 | pSG61 with uORF1 penultimate codon mutated to GAA | Average |
| pSG81 | pSG61 with uORF1 penultimate codon mutated to CGC | Average |
| pSG82 | pSG61 with uORF1 penultimate codon mutated to GTC | Average |
| pSG83 | pSG61 with uORF1 penultimate codon mutated to GGG | Average |
| pSG84 | pSG61 with uORF1 penultimate codon mutated to CCG | Average |
| pSG85 | pSG61 with uORF1 penultimate codon mutated to CGG | Average |
| pSG86 | pSG61 with uORF1 penultimate codon mutated to CCC | Average |
| pSG87 | pSG61 with uORF1 penultimate codon mutated to TCG | Average |
| pSG88 | pSG61 with uORF1 penultimate codon mutated to AGG | Average |
| pSG89 | pSG61 with uORF1 penultimate codon mutated to CCA | Average |
| pSG61 (2) (uORF3-only) | low copy *URA3* *GCN4-lacZ* plasmid containing the *GCN4* leader with uORF3 only at its original position^5^ | N/A |
| p209 (uORF1-only) | low copy *URA3* *GCN4-lacZ* plasmid containing the *GCN4* leader with uORF1 only at its original position^6^ | Average |

**Supplementary REFERENCES**

1. Mueller PP, Harashima S, Hinnebusch AG. A segment of *GCN4* mRNA containing the upstream AUG codons confers translational control upon a heterologous yeast transcript. *Proc Natl Acad Sci USA* **84**, 2863-2867 (1987).

2. Longtine MS*, et al.* Additonal modules for versatile and economical PCR-based gene deletion and modification in *Saccharomyces cerevisiae*. *Yeast* **14**, 953-961 (1998).

3. Carter Z, Delneri D. New generation of loxP-mutated deletion cassettes for the genetic manipulation of yeast natural isolates. *Yeast* **27**, 765-775 (2010).

4. Gunisova S, Beznoskova P, Mohammad MP, Vlckova V, Valasek LS. In-depth analysis of cis-determinants that either promote or inhibit reinitiation on GCN4 mRNA after translation of its four short uORFs. *RNA* **22**, 542-558 (2016).

5. Gunisova S, Valasek LS. Fail-safe mechanism of GCN4 translational control-uORF2 promotes reinitiation by analogous mechanism to uORF1 and thus secures its key role in GCN4 expression. *Nucleic Acids Res* **42**, 5880-5893 (2014).

6. Grant CM, Hinnebusch AG. Effect of sequence context at stop codons on efficiency of reinitiation in *GCN4* translational control. *Mol Cell Biol* **14**, 606-618 (1994).
