# Supplementary material for: Differential effects of 40S ribosome recycling factors on reinitiation at regulatory uORFs in GCN4 mRNA are not dictated by their roles in bulk 40S recycling
Source: Commun Biol. 2024 Sep 4;7:1083. doi: 10.1038/s42003-024-06761-x (PMC11375166; doi:10.1038/s42003-024-06761-x)
Supplement: Supplementary file 5 — Supplementary Data 3 [file 42003_2024_6761_MOESM5_ESM.docx]

**Supplementary Data 3. Synthetized DNA inserts used in this study.**

This table lists the sequences of DNA fragments used for cloning that were synthesized through GeneArt Gene Synthesis (Thermo Fisher Scientific) or by LifeSct LLC. The table is formatted as follows: Insert name (construct it was used for); Construct sequence.

| Insert (construct): | Sequence: |
| --- | --- |
| MFL (pKJ17) | GAGCGCGACGTAATACGACTCACTATAGGGCGAATTGGCGGAAGGCCGTCAAGGCCGCATCAAGGGCATCGGTCGACGGGGAATAAAGTGCATGAGCATACATCTTGAAAAAAAAAGATGAAAAATTTCCGACTTTAAATACGGAAGATAAATACTCCAACCTTTTTTTCCAATTCCGAAATTTTAGTCTTCTTTAAAGAAGTTTCGGCTCGCTGTCTTACCTTTTAAAATCTTCTACTTCTTGACAGTACTTATCTTCTTATATAATAGATATACAAAACAAAACAAAACAAAAACTCACAACACAGGTTACTCTCCCCCCTAAATTCAAATTTTTTTTGCCCATCAGTTTCACTAGCGAATTATACAACTCACCAGCCACACAGCTCACTCATCTACTTCGCAATCAAAACAAAATATTTTATTTTAGTTCAGTTTATTAAGTTATTATCAGTATCGTATTAAAAAATTAAAGATCATTGAAAAAAGCTTGCTAAACCGATTATATTTTGTTTTTAAAGTAGATTATTATTAGAAAATTATTAAGAGAATTCTGTGTTAAATTTATTGAAAGAGAAAATTTATTTTCCCTTATTAATTAAAGTCCTTTACTTTTTTTGAAAACTGTCAGTTTTTTGAAGAGTTATTTGTTTTGTTACCAATTGCTATCAGGTACCCGTAGAATTTTATTCAAGATGTTTCTGTAACGGTTACCTTTCTGTCAAACTGGGCCTCATGGGCCTTCCGCTCACTGCCCGCTTTCCAGTCGGGAAACCTGTCGTGCCA |
| MFM (pKJ23) | GAGCGCGACGTAATACGACTCACTATAGGGCGAATTGGCGGAAGGCCGTCAAGGCCGCATCAAGGGCATCGGTCGACGGGGAATAAAGTGCATGAGCATACATCTTGAAAAAAAAAGATGAAAAATTTCCGACTTTAAATACGGAAGATAAATACTCCAACCTTTTTTTCCAATTCCGAAATTTTAGTCTTCTTTAAAGAAGTTTCGGCTCGCTGTCTTACCTTTTAAAATCTTCTACTTCTTGACAGTACTTATCTTCTTATATAATAGATATACAAAACAAAACAAAACAAAAACTCACAACACAGGTTACTCTCCCCCCTAAATTCAAATTTTTTTTGCCCATCAGTTTCACTAGCGAATTATACAACTCACCAGCCACACAGCTCACTCATCTACTTCGCAATCAAAACAAAATATTTTATTTTAGTTCAGTTTATTAAGTTATTATCAGTATCGTATTAAAAAATTAAAGATCATTGAAAAAAGCTTGCTAAACCGATTATATTTTGTTTTTAAAGTAGATTATTATTAGAAAATTATTAAGAGAATTCTGTGTTAAATTTATTGAAAGAGAAAATTTATTTTCCCTTATTAATTAAAGTCCTTTACTTTTTTTGAAAACTGTCAGTTTTTTGAAGAGTTATTTGTTTTGTTACCAATTGCTATCAGGTACCCGTAGAATTTTATTCAAGATGTTTATGTAACGGTTACCTTTCTGTCAAACTGGGCCTCATGGGCCTTCCGCTCACTGCCCGCTTTCCAGTCGGGAAACCTGTCGTGCCA |
| MFY (pKJ20) | GAGCGCGACGTAATACGACTCACTATAGGGCGAATTGGCGGAAGGCCGTCAAGGCCGCATCAAGGGCATCGGTCGACGGGGAATAAAGTGCATGAGCATACATCTTGAAAAAAAAAGATGAAAAATTTCCGACTTTAAATACGGAAGATAAATACTCCAACCTTTTTTTCCAATTCCGAAATTTTAGTCTTCTTTAAAGAAGTTTCGGCTCGCTGTCTTACCTTTTAAAATCTTCTACTTCTTGACAGTACTTATCTTCTTATATAATAGATATACAAAACAAAACAAAACAAAAACTCACAACACAGGTTACTCTCCCCCCTAAATTCAAATTTTTTTTGCCCATCAGTTTCACTAGCGAATTATACAACTCACCAGCCACACAGCTCACTCATCTACTTCGCAATCAAAACAAAATATTTTATTTTAGTTCAGTTTATTAAGTTATTATCAGTATCGTATTAAAAAATTAAAGATCATTGAAAAAAGCTTGCTAAACCGATTATATTTTGTTTTTAAAGTAGATTATTATTAGAAAATTATTAAGAGAATTCTGTGTTAAATTTATTGAAAGAGAAAATTTATTTTCCCTTATTAATTAAAGTCCTTTACTTTTTTTGAAAACTGTCAGTTTTTTGAAGAGTTATTTGTTTTGTTACCAATTGCTATCAGGTACCCGTAGAATTTTATTCAAGATGTTTTACTAACGGTTACCTTTCTGTCAAACTGGGCCTCATGGGCCTTCCGCTCACTGCCCGCTTTCCAGTCGGGAAACCTGTCGTGCCA |
| MFA (pKJ21) | GAGCGCGACGTAATACGACTCACTATAGGGCGAATTGGCGGAAGGCCGTCAAGGCCGCATCAAGGGCATCGGTCGACGGGGAATAAAGTGCATGAGCATACATCTTGAAAAAAAAAGATGAAAAATTTCCGACTTTAAATACGGAAGATAAATACTCCAACCTTTTTTTCCAATTCCGAAATTTTAGTCTTCTTTAAAGAAGTTTCGGCTCGCTGTCTTACCTTTTAAAATCTTCTACTTCTTGACAGTACTTATCTTCTTATATAATAGATATACAAAACAAAACAAAACAAAAACTCACAACACAGGTTACTCTCCCCCCTAAATTCAAATTTTTTTTGCCCATCAGTTTCACTAGCGAATTATACAACTCACCAGCCACACAGCTCACTCATCTACTTCGCAATCAAAACAAAATATTTTATTTTAGTTCAGTTTATTAAGTTATTATCAGTATCGTATTAAAAAATTAAAGATCATTGAAAAAAGCTTGCTAAACCGATTATATTTTGTTTTTAAAGTAGATTATTATTAGAAAATTATTAAGAGAATTCTGTGTTAAATTTATTGAAAGAGAAAATTTATTTTCCCTTATTAATTAAAGTCCTTTACTTTTTTTGAAAACTGTCAGTTTTTTGAAGAGTTATTTGTTTTGTTACCAATTGCTATCAGGTACCCGTAGAATTTTATTCAAGATGTTTGCGTAACGGTTACCTTTCTGTCAAACTGGGCCTCATGGGCCTTCCGCTCACTGCCCGCTTTCCAGTCGGGAAACCTGTCGTGCCA |
| uORF1 st-st fragment (pKP78) | GCTCTCAAGGGCATCGGTCGACGGGGAATAAAGTGCATGAGCATACATCTTGAAAAAAAA  AGATGAAAAATTTCCGACTTTAAATACGGAAGATAAATACTCCAACCTTTTTTTCCAATT  CCGAAATTTTAGTCTTCTTTAAAGAAGTTTCGGCTCGCTGTCTTACCTTTTAAAATCTTC  TACTTCTTGACAGTACTTATCTTCTTATATAATAGATATACAAAACAAAACAAAACAAAA  ACTCACAACACAGGTTACTCTCCCCCCTAAATTCAAATTTTTTTTGCCCATCAGTTTCAC  TAGCGAATTATACAACTCACCAGCCACACAGCTCACTCATCTACTTCGCAATCAAAACAA  AATATTTTATTTTAGTTCAGTTTATTAAGTTATTATCAGTATCGTATTAAAAAATTAAAG  ATCATTGAAAAATGTAAACCGATTATATTTTGTTTTTAAAGTAGATTATTATTAGAAAAT  TATTAAGAGAATTCTGTGTTAAATTTATTGAAAGAGAAAATTTATTTTCCCTTATTAATT  AAAGTCCTTTACTTTTTTTGAAAACTGTCAGTTTTTTGAAGAGTTATTTGTTTTGTTACC  AATTGCTATCAGGTACCCGTAGAATTTTATTCAAGATCTTTCCGTAACGGTTACCTTTCT  GTCAAATTATC |
| uORF3 st-st fragment (pKP76) | CAAGGGCATCGGTCGACGGGGAATAAAGTGCATGAGCATACATCTTGAAAAAAAAAGATG  AAAAATTTCCGACTTTAAATACGGAAGATAAATACTCCAACCTTTTTTTCCAATTCCGAA  ATTTTAGTCTTCTTTAAAGAAGTTTCGGCTCGCTGTCTTACCTTTTAAAATCTTCTACTT  CTTGACAGTACTTATCTTCTTATATAATAGATATACAAAACAAAACAAAACAAAAACTCA  CAACACAGGTTACTCTCCCCCCTAAATTCAAATTTTTTTTGCCCATCAGTTTCACTAGCG  AATTATACAACTCACCAGCCACACAGCTCACTCATCTACTTCGCAATCAAAACAAAATAT  TTTATTTTAGTTCAGTTTATTAAGTTATTATCAGTATCGTATTAAAAAATTAAAGATCAT  TGAAAAAAGCTTGCTAAACCGATTATATTTTGTTTTTAAAGTAGATTATTATTAGAAAAT  TATTAAGAGAATTCTGTGTTAAATTTATTGAAAGAGAAAATTTATTTTCCCTTATTAATT  AAAGTCCTTTACTTTTTTTGAAAACTGTCAGTTTTTTGAAGAGTTATTTGTTTTGTTACC  AATTGCTATCATGTAGAATTTTATTCAAGATCTTTCCGTAACGGTTACCTTTCTGTCAAA |
| uORF4 st-st fragment (pKP77) | CAAGGGCATCGGTCGACGGGGAATAAAGTGCATGAGCATACATCTTGAAAAAAAAAGATG  AAAAATTTCCGACTTTAAATACGGAAGATAAATACTCCAACCTTTTTTTCCAATTCCGAA  ATTTTAGTCTTCTTTAAAGAAGTTTCGGCTCGCTGTCTTACCTTTTAAAATCTTCTACTT  CTTGACAGTACTTATCTTCTTATATAATAGATATACAAAACAAAACAAAACAAAAACTCA  CAACACAGGTTACTCTCCCCCCTAAATTCAAATTTTTTTTGCCCATCAGTTTCACTAGCG  AATTATACAACTCACCAGCCACACAGCTCACTCATCTACTTCGCAATCAAAACAAAATAT  TTTATTTTAGTTCAGTTTATTAAGTTATTATCAGTATCGTATTAAAAAATTAAAGATCAT  TGAAAAAAGCTTGCTAAACCGATTATATTTTGTTTTTAAAGTAGATTATTATTAGAAAAT  TATTAAGAGAATTCTGTGTTAAATTTATTGAAAGAGAAAATTTATTTTCCCTTATTAATT  AAAGTCCTTTACTTTTTTTGAAAACTGTCAGTTTTTTGAAGAGTTATTTGTTTTGTTACC  AATTGCTATCAGGTACCCGTAGAATTTTATTCAAGATGTAACGGTTACCTTTCTGTCAAA |
| *Sal*I*-Bst*EII fragment of pSG61 with WT uORF1 underlined. For pSG61-pSG89, the third (TGC) codon is replaced with other codons. | GTCGACCCCGTCCTGTGGATCTTCGGGGAATAAAGTGCATGAGCATACATCTTGAAAAAAAAAGATGAAAAATTTCCGACTTTAAATACGGAAGATAAATACTCCAACCTTTTTTTCCAATTCCGAAATTTTAGTCTTCTTTAAAGAAGTTTCGGCTCGCTGTCTTACCTTTTAAAATCTTCTACTTCTTGACAGTACTTATCTTCTTATATAATAGATATACAAAACAAAACAAAACAAAAACTCACAACACAGGTTACTCTCCCCCCTAAATTCAAATTTTTTTTGCCCATCAGTTTCACTAGCGAATTATACAACTCACCAGCCACACAGCTCACTCATCTACTTCGCAATCAAAACAAAATATTTTATTTTAGTTCAGTTTATTAAGTTATTATCAGTATCGTATTAAAAAATTAAAGATCATTGAAAAATGGCTTGCTAAACCGATTATATTTTGTTTTTAAAGTAGATTATTATTAGAAAATTATTAAGAGAATTCTGTGTTAAATTTATTGAAAGAGAAAATTTATTTTCCCTTATTAATTAAAGTCCTTTACTTTTTTTGAAAACTGTCAGTTTTTTGAAGAGTTATTTGTTTTGTTACCAATTGCTATCAGGTACCCGTAGAATTTTATTCAAGAGGTTTCCGTAACGGTTACCTTTCTGTCAAATTATCCAGGTTTACTCGCCAATAAAAATTTCCCTATACTATCATTAATTAAATCATTATTATTACTAAAGTTTTGTTTACC |
